# Cross-Species Transmission of Coronaviruses in Humans and Domestic Mammals, What Are the Ecological Mechanisms Driving Transmission, Spillover, and Disease Emergence?

**DOI:** 10.3389/fpubh.2021.717941

**Published:** 2021-09-30

**Authors:** Nicole Nova

**Affiliations:** Department of Biology, Stanford University, Stanford, CA, United States

**Keywords:** coronavirus, COVID-19, cross-species transmission, host range, MERS, One Health, SARS, spillover

## Abstract

Coronaviruses cause respiratory and digestive diseases in vertebrates. The recent pandemic, caused by the novel severe acute respiratory syndrome (SARS) coronavirus 2, is taking a heavy toll on society and planetary health, and illustrates the threat emerging coronaviruses can pose to the well-being of humans and other animals. Coronaviruses are constantly evolving, crossing host species barriers, and expanding their host range. In the last few decades, several novel coronaviruses have emerged in humans and domestic animals. Novel coronaviruses have also been discovered in captive wildlife or wild populations, raising conservation concerns. The evolution and emergence of novel viruses is enabled by frequent cross-species transmission. It is thus crucial to determine emerging coronaviruses' potential for infecting different host species, and to identify the circumstances under which cross-species transmission occurs in order to mitigate the rate of disease emergence. Here, I review (broadly across several mammalian host species) up-to-date knowledge of host range and circumstances concerning reported cross-species transmission events of emerging coronaviruses in humans and common domestic mammals. All of these coronaviruses had similar host ranges, were closely related (indicative of rapid diversification and spread), and their emergence was likely associated with high-host-density environments facilitating multi-species interactions (e.g., shelters, farms, and markets) and the health or well-being of animals as end- and/or intermediate spillover hosts. Further research is needed to identify mechanisms of the cross-species transmission events that have ultimately led to a surge of emerging coronaviruses in multiple species in a relatively short period of time in a world undergoing rapid environmental change.

## Introduction

Coronaviruses (CoVs) cause respiratory and digestive diseases in humans and other animals, and are responsible for several emerging diseases. The severe acute respiratory syndrome (SARS) outbreak in 2002–2003 resulted in 8,422 human cases and 916 deaths in 33 countries (1). In 2012, Middle East respiratory syndrome (MERS) emerged, and over time has resulted in over 2,500 human cases and 866 deaths in 27 countries (2, 3). As of mid-2021, the novel coronavirus disease 2019 (COVID-19) pandemic has resulted in 4.2 million human deaths and 196.2 million cases in 221 countries and territories (4). Other animals have also been affected by these and other emerging coronaviruses, all of which resulted from cross-species transmission, and demonstrate the serious threat coronaviruses can pose to humans and other animals globally.

Named after their crown-shaped spike surface proteins, coronaviruses are enveloped, positive-sense single-stranded RNA viruses that belong to the family *Coronaviridae*, subfamily *Orthocoronavirinae* (5, 6). They split into four genera: *Alphacoronavirus, Betacoronavirus, Deltacoronavirus*, and *Gammacoronavirus* (5). The first two genera infect mammals primarily, whereas *Gammacoronaviruses* infect birds, and *Deltacoronaviruses* infect both mammals and birds (7). Coronaviruses further split into species; however, they exist as quasispecies due to the rapid evolution driven by their high mutation rates and homologous RNA recombination (8). Coronaviruses have the largest genomes (26.4–31.7 kb) of all known RNA viruses; thus, their genomes are especially prone to accumulation of mutations and recombined segments over time, which contributes to their diverse host range and potential for disease emergence (9).

Bats are considered reservoirs for most *Alpha-* and *Betacoronaviruses*, while wild birds are probable reservoirs for *Gamma-* and *Deltacoronaviruses* (10). Coronavirus spillover from reservoirs to other species, and subsequent cross-species transmission, is primarily mediated by recombination in the receptor-binding domain (RBD) of the spike protein (S) gene (11). The receptor-binding domain enables coronaviruses to infect hosts by binding to a host receptor, e.g., angiotensin-converting enzyme 2 (ACE2) in the case of SARS coronaviruses, for cell entry (7, 12, 13). Although research has revealed reservoirs and molecular mechanisms enabling cross-species transmission, and that viral evolution is facilitated by frequent cross-species transmission events (14), less is known about the environments favoring emerging coronavirus evolution in non-reservoir hosts.

Agriculture and industrialization expanded the global abundance of humans and domestic mammals (i.e., livestock and pets). Today, their combined biomass makes up 96% of all mammalian biomass on Earth (15). This may be the primary reason for disease emergence in humans and other animals (16). To help curb coronavirus disease emergence, it is important to identify current host ranges of existing coronaviruses in humans and domestic animals, and the circumstances associated with their cross-species transmission.

This review provides an updated succinct summary of known host ranges and cross-species transmissions of recently emerged coronaviruses in humans and domestic mammals. Moreover, I discuss commonalities among the ecological circumstances related to spillover and emergence of several coronaviruses in various mammalian hosts, and how these may inform One Health interventions for preventing disease emergence.

## Emerging Human Coronaviruses

There are seven known human coronaviruses: the *Betacoronaviruses* SARS-CoV-1, MERS-CoV, and SARS-CoV-2, which caused SARS, MERS, and COVID-19, respectively, and the *Alphacoronaviruses* NL63 and 229E and *Betacoronaviruses* OC43 and HKU1, which cause the common cold in humans (17). The latter four may not be labeled as recently emerging coronaviruses, although they have spilled over at some point in the past. Bats are considered reservoirs for NL63 and 229E, whereas rodents are putative reservoirs for OC43 and HKU1 (17–19). NL63 possibly emerged several hundred years ago from recombination between ancestors to 229E in hipposiderid bats and coronaviruses circulating in African trident bats (19, 20). Based on phylogenetic analyses, cattle and camelids have been identified as probable intermediate spillover hosts for OC43 and 229E emergence one and two centuries ago, respectively (17, 18, 20). The bovine-to-human spillover that led to OC43 emergence likely coincided with a pandemic in 1890 (17, 21, 22). Indeed, OC43 and bovine coronavirus share 96% global nucleotide identity (23). Finally, extant lineages of HKU1 trace their most recent common ancestor to the 1950s, when it possibly spilled over from rodents (20).

Next, this section covers plausible spillover events—from reservoirs to humans via potential intermediate host species—that generated the recent SARS-CoV-1, MERS-CoV, and SARS-CoV-2, and their cross-species transmission potential.

### SARS-CoV-1

Severe acute respiratory syndrome emerged in Guangdong, China, and caused the devastating 2000–2003 outbreak in several countries (1). Successful efforts curbed the epidemic: only a few cases occurred in late 2003 and early 2004 (24). There have been no known SARS-CoV-1-related cases since.

Based on genetic and epidemiologic investigations, the first SARS-CoV-1-infected individuals likely contracted the virus from masked palm civets or other wildlife in wet markets (24–27). Civet isolates revealed ongoing adaptation, suggesting that they were not reservoir hosts, but intermediate spillover hosts that contracted the virus from horseshoe bats (26–30). Substantial evidence confirms bats as SARS reservoirs (26, 28, 29, 31, 32).

Wildlife samples from a market in Shenzhen revealed that SARS-CoV-1 shared 99.8% nucleotide identity with isolates from civets and a raccoon dog, and that a ferret badger had seroconverted against SARS-CoV-1 (24, 26). Initial human cases reported direct or indirect contact with these animals via handling, killing, meat serving, or residing near wet markets (33). Surveys showed that animal (especially civet) traders, although asymptomatic, had disproportionately high seroconversion against SARS-CoV-1, suggesting they have been exposed to SARS-CoV-related viruses for several years before the SARS epidemic (24, 26). Intermediate spillover hosts were not necessarily required for the evolution of SARS-CoV-1, since a bat SARS-like coronavirus is able to bind to ACE2 in humans and civets for cell entry (34). Nonetheless, civets may have amplified the virus and brought it closer to humans (35).

Additional mammals are susceptible to SARS-CoV-1 infection. Cats, ferrets, guinea pigs, golden hamsters, common marmosets, grivets, and cynomolgus and rhesus macaques can be infected under experimental inoculation, seroconvert, and display similar pathological signs as humans, and the monkeys and guinea pigs usually display mild clinical signs, while cats and golden hamsters show no clinical signs (36–44). In two studies, inoculated ferrets only exhibited signs of lethargy (36, 37). Furthermore, cats and ferrets can shed SARS-CoV-1 and transmit the virus within each species (36). Cats have also been naturally infected by SARS-CoV-1 in an apartment block where residents had SARS, suggesting possible human-to-cat transmission (36). Although swine are susceptible to SARS-CoV-1 both experimentally and naturally, viral replication in (and shedding from) swine is poor (45–47). Mice and poultry are not susceptible to SARS-CoV-1 infection (45, 48, 49). Thus, SARS-CoV-1 was not uniquely adapted to humans, yet likely restricted to mammals.

### MERS-CoV

Middle East respiratory syndrome cases are still being reported since it became endemic in the Arabian peninsula. Middle East respiratory syndrome does sporadically spread to other parts of the world, although with limited human-to-human transmission (50, 51). Most outbreaks originate from independent spillover events.

Bats are putative reservoirs for MERS, while dromedary camels and other camelids are intermediate spillover hosts (52–54). Although rare, camel-to-human transmission does occur (51, 55). Infected camels shed MERS-CoV via bodily fluids, especially nasal secretions, and exhibit sneezing, coughing, fever, and loss of appetite (56, 57). Camel care-takers or consumers of camel products are at risk of contracting MERS-CoV (51). People in direct or indirect contact with camels have disproportionately high seroconversion against MERS-CoV (58). Surveys from 2010 to 2013 in Saudi Arabia show that 90% of 310 and 74% of 203 camels were MERS-CoV seropositive (59, 60). Historical seropositive samples and phylogenetic analyses suggest that MERS-like coronaviruses have been circulating in camels for at least a few decades before MERS recently emerged in humans (52, 60–63). Camel markets with both live and dead animals are believed to serve as hotspots for MERS-CoV transmission (64).

MERS-CoV may infect additional species. Rhesus macaques, common marmosets, swine, llamas, rabbits, and alpacas have been infected experimentally, and the monkeys developed mild-to-moderate and moderate-to-severe disease, respectively, swine and llamas displayed rhinorrhea, while rabbits and alpacas showed no clinical signs, although alpacas shed MERS-CoV and transmitted it within its species (65–68). A virological survey found MERS-CoV in sheep, goats, donkeys, and a cow, but not in buffaloes, mules, or horses (69). A serological study confirms that equids might not be susceptible to MERS-CoV infection, although *in vitro* inoculation suggests otherwise (70). However, in an experimental inoculation study, sheep and horses did not show evidence of viral replication or seroconversion (68). Mice, golden hamsters, ferrets, and poultry are not considered susceptible to MERS-CoV infection, mainly because of their low host receptor homology with that of the MERS-CoV-susceptible species (67, 71).

### SARS-CoV-2

The current COVID-19 pandemic was initially reported in Wuhan, China in 2019 (72, 73), although the origin of its pathogen, SARS-CoV-2, is still unclear. Its ancestor probably originated in bats, since SARS-CoV-2 is most closely related to the 2013 and 2019 isolates from horseshoe bats in Yunnan, China at the genome level, although not at the RBD level, suggesting neither might bind to human ACE2, and are thus not immediate ancestors of SARS-CoV-2 (72, 74, 75).

Conversely, isolates (pangolin-CoVs) from smuggled and diseased pangolins in Guangdong (2018–2019) are closely related to SARS-CoV-2 in the RBD region (76–80). Molecular binding simulations show that S proteins of SARS-CoV-2 and pangolin-CoVs can potentially recognize ACE2 in both humans and pangolins, suggesting possible pangolin-to-human spillover (76, 77). However, because pangolin-CoVs (including strains from Guangxi) are not the closest relatives to SARS-CoV-2 at the genome level, they are likely not direct ancestors of SARS-CoV-2 (76, 78, 79). Nevertheless, a 2019 pangolin-CoV isolate from Guangdong displayed high genome-wide similarity with both SARS-CoV-2 and SARS-CoV-2's closest relative (from bats), suggesting SARS-CoV-2 may have originated from recombination among coronaviruses present in bats and other wildlife (76, 77, 79, 81).

Like SARS-CoV-1, SARS-CoV-2 infects species with high ACE2 homology. Cats, ferrets, golden hamsters, tree shrews, common marmosets, grivets, and cynomolgus and rhesus macaques have been infected with SARS-CoV-2 experimentally, shed the virus, and displayed similar or milder clinical and pathological signs as humans, although cats may not show signs of disease (82–91). Conversely, dogs have low susceptibility to SARS-CoV-2, and show lack of clinical signs or dog-to-dog transmission, possibly due to their low levels of ACE2 in the respiratory tract (82, 91–93). Yet, cat-to-cat, ferret-to-ferret, hamster-to-hamster, and bat-to-bat transmission of SARS-CoV-2 have been confirmed experimentally (82, 90, 91, 94). However, mice, swine, and poultry are not susceptible to SARS-CoV-2 infection (49, 71, 82).

Accumulating evidence supports naturally occurring human-to-cat SARS-CoV-2 transmission, such as multiple reports worldwide of SARS-CoV-2-positive cats from confirmed or suspected SARS-CoV-2-positive owners (95). Natural human-to-dog transmission may be possible, as was confirmed by seroconversion and SARS-CoV-2 presence in two out of 15 dogs in close contact with COVID-19 patients, where the viral sequences from each dog-and-owner pair were identical (92). Serological and virological surveys, conducted several months after the pandemic started, indicate that SARS-CoV-2 prevalence is much lower in pet and street cats and dogs than in humans, even if pet owners had suspected or confirmed SARS-CoV-2 infection (96–100). Thus, cats and dogs can get infected under natural conditions, but rarely. However, certain environments might amplify natural infections and cross-species transmission. Human-to-mink, mink-to-mink, and mink-to-human transmission of SARS-CoV-2 have occurred on fur farms in several countries (95, 101–104). SARS-CoV-2 has also been transmitted to tigers, lions, and gorillas in zoos, raising concern for wildlife conservation (105).

Apart from the mink farm outbreaks, evidence so far suggests limited SARS-CoV-2 maintenance in domestic mammals or risk for secondary zoonoses (104). However, the panzootic potential of SARS-CoV-2 necessitates expanding veterinary surveillance (104, 106), especially if domestic and/or wild animals were to maintain SARS-CoV-2 as the human population undergoes vaccination, making COVID-19 control more difficult.

## Emerging Coronaviruses in Domestic Mammals

Since the advent of agriculture (~8,000 BC), several spillover events have led to the emergence of novel pathogens in humans and domesticated animals (16). Genetic analyses place the common ancestor to all known coronaviruses at around 8,000 BC, and those of each genus at around 2,400–3,300 BC (10). Like humans, domestic mammals have been experiencing an increasing rate of novel coronavirus emergence, especially within the last century.

Bovine coronavirus (BCoV) likely emerged from rodent-CoVs around 1400 AD (17, 107). Bovine coronavirus is transmitted via the fecal–oral route, causing bloody diarrhea and respiratory infections in cattle (108–110). Bovine coronavirus-like viruses have also been detected in other domestic and wild ruminants (108). Bovine coronavirus can infect dogs experimentally, although subclinically (111). Turkeys show clinical signs of enteritis when infected with BCoV experimentally, but chickens are not susceptible (112). Equine-CoV, discovered in 1999, plausibly also descended from BCoV and causes enteritis in horses (113–115).

There are two dog coronaviruses: an *Alphacoronavirus* called canine enteric coronavirus (CCoV), transmitted fecal-orally, with serotypes CCoV-I and CCoV-II, and a *Betacoronavirus* called canine respiratory coronavirus (CRCoV), which causes kennel cough (116). Canine respiratory coronavirus was discovered in 2003 from a kennel outbreak (117). It was later also detected in samples from 1996 (118). It is closely related to BCoV and OC43, and genetic analyses suggest that CRCoV arose from a recent host-species shift of BCoV from bovine to canine hosts (117, 119).

Canine enteric coronavirus was first isolated from an outbreak in military dogs in 1971 (116). Initially, CCoV infections were believed to be restricted to the enteric tract causing mild diarrheal disease (120), but an increasing number of lethal pantropic infections suggests that CCoV is responsible for an emerging infectious disease in canines (116). There are three proposed subtypes of CCoV-II: original CCoV-IIa, recombinant CCoV-IIb, and CCoV-IIc (116). The two biotypes of CCoV-IIa have different tissue tropism and pathogenicity: “classical” CCoV-IIa is restricted to the small intestine causing enteritis, but the emerging “pantropic” CCoV-IIa causes leukopenia and is often fatal (116, 121). In 2019, an Asian pantropic CCoV-IIa strain was also isolated from a wolf in Italy (122), suggesting spillover to wildlife of imported strains (123). Cats and swine are also susceptible to CCoV (124–126).

There are six porcine coronaviruses: four *Alphacoronaviruses*, transmissible gastroenteritis virus (TGEV), porcine respiratory coronavirus (PRCoV), porcine epidemic diarrhea virus (PEDV), and swine acute diarrhea syndrome coronavirus (SADS-CoV), one *Betacoronavirus*, porcine haemagglutinating encephalomyelitis virus (PHEV), and one *Deltacoronavirus*, porcine deltacoronavirus (PDCoV) (127). Transmissible gastroenteritis virus, PEDV, SADS-CoV, and PDCoV cause severe enteritis that are fatal in piglets, PHEV causes digestive and/or neurological disease, and PRCoV causes mild respiratory disease (127).

Transmissible gastroenteritis virus, discovered in 1946 (128), likely emerged from CCoV-II (129), and its less virulent descendent PRCoV was identified in 1984 (130). Porcine haemagglutinating encephalomyelitis virus, first described in 1957, likely descended from BCoV (127). Porcine epidemic diarrhea virus emerged in the 1970s in Europe and Asia, likely from bat-CoVs, and was introduced in North America in 2013 after a new PEDV strain emerged in China in 2010 (131–134). A serological study indicates that PEDV subsequently spilled over from domestic to feral swine populations in the US (135). Porcine deltacoronavirus was first detected in swine samples from 2009 in Hong Kong (10, 132). In 2014, PDCoV caused the first-reported outbreaks in USA and South Korea (136, 137). It was proposed that the virus' ancestor originated from recombination between sparrow-CoV and bulbul-CoV (138). Porcine deltacoronavirus is most closely related to *Deltacoronaviruses* sampled from Asian leopard cats and ferret badgers in Guangdong and Guangxi markets (the first documented cases of *Deltacoronaviruses* in mammals) (139), suggesting that these species could have acted as intermediates for interspecies PDCoV spillover (140). In 2016, SADS outbreaks emerged in Guangdong with evidence strongly suggesting bat-to-swine spillover origin (141).

There is one coronavirus that primarily infects cats: feline coronavirus (FCoV). This *Alphacoronavirus* exists in two serotypes: FCoV-I and FCoV-II (142). Both cause digestive diseases and are transmitted fecal-orally. FCoV-I is the most common type, but less virulent than FCoV-II (143, 144). Comparative sequence studies indicate FCoV-I is genetically similar to CCoV-I, and FCoV-II emerged from recombination between FCoV-I and CCoV-II (121, 142, 145, 146). Conceivably, FCoV-I and CCoV-I evolved from a common ancestor, while CCoV-II and FCoV-II arose as more virulent recombinants (129). For each serotype, there are two biotypes with different pathogenicity: feline enteric coronavirus (FECV) and feline infectious peritonitis virus (FIPV). Feline enteric coronavirus usually causes mild diarrhea, whereas feline infectious peritonitis (FIP) is lethal. Feline infectious peritonitis virus evolves from FECV via within-host mutations in the S gene that alter cell tropism, and emerges during persistent infection of FECV (142, 147). However, a novel FIPV strain may have been transmitted horizontally (144). In 2004, a disease resembling FIP was also discovered in ferrets caused by an emerging ferret systemic coronavirus, a decade after the first and less virulent ferret coronavirus (enteric) was discovered (148). Feline infectious peritonitis likely emerged in the late 1950s, within a decade after the first TGE cases in swine in USA (128, 149). Thus, FCoV is closely related to TGEV and CCoV, and recombinants among all three have emerged (150–152), probably because all three can cross-infect cats, swine, and dogs (125, 151, 153–155).

## Discussion

**Figure 1:**
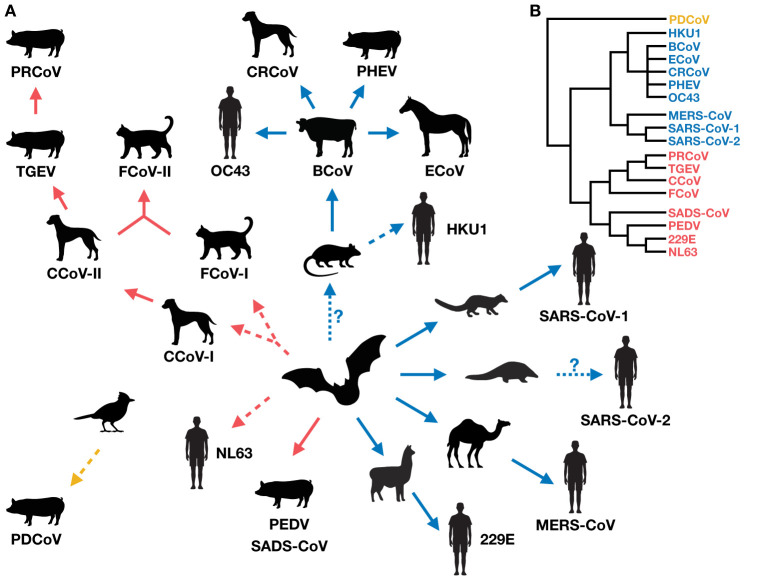
**(A)** The evolution and radiation of coronaviruses in humans and domestic mammals (via potential wild intermediate spillover host species). The radiation suggests there could be a vicious cycle of coronavirus emergence, whereby newly emerged viruses in new hosts increase the likelihood of producing more new recombinants. Red, blue and yellow arrows indicate the direction of spillover of coronavirus emergence for *Alphacoronaviruses, Betacoronaviruses*, and *Deltacoronaviruses*, respectively. Solid arrows represent direct (confirmed or suspected) coronavirus transmission between host species (although indirect transmission via an unidentified intermediate host is not excluded), and dashed arrows represent suspected indirect transmission via an unidentified intermediate host (although direct transmission is not excluded) (10, 17, 104, 127, 139, 141, 156). Dotted arrows with a question mark indicate uncertain spillover events. **(B)** A simplified phylogeny of the coronaviruses covered in this review, drawn from published findings (5, 129, 157).

Coronaviruses in humans and domestic animals are closely related (Figure 1), and have emerged recently and at an increasing rate. The circumstances associated with their emergence are high-animal-density environments that favor interspecies interactions, such as kennels, shelters, farms, and markets (Table 1), which increase disease prevalence and promote cross-species transmission. Indeed, studies show that seroprevalence of CCoV is higher in kennels compared to the rest of the dog population, and shelters co-housing dogs with cats harbor recombinant canine-feline coronaviruses (116, 151, 153, 159). Further, commercial agriculture has led to large numbers of domestic animals living in close proximity to humans, possibly driving the emergence of OC43 from cattle, and 229E and MERS from camelids.

**Table 1:** First reported outbreaks and probable host species involved in the cross-species transmission events of recently emerging coronaviruses (or new virulent strains of re-emerging coronaviruses) in humans and domestic mammals covered in this review.

**Primary host**	**Emerging coronavirus** **(***or new virulent strain***)**	**Year and location of** **first reported cases**	**Intermediate spillover host or host of viral predecessor**	**Potential reservoir host**	**Environment associated with emergence**	**References**
Human	SARS-CoV-1	2002 Guangdong, China	Masked palm civet (*Paguma larvata*)	Bat (*Rhinolophus* spp.)	Wet market	(1, 24–30, 156)
	MERS-CoV	2012 Saudi Arabia	Dromedary camel (*Camelus dromedarius*)	Bat (*Taphozous perforatus, Rhinopoma hardwickii* and *Pipistrellus kuhlii*)	Camel farm and market	(2, 3, 52–54, 64, 156)
	SARS-CoV-2	2019 Wuhan, China	Malayan pangolin (*Manis javanica*)?	Bat (*Rhinolophus* spp.)	Wildlife trade and/or wet market?	(72, 73, 75, 79)
Pig	Porcine epidemic diarrhea virus (PEDV)	1978 Belgium	Unknown	Bat (*Scotophilus kuhlii*)	Swine farm	(156)
	*New virulent PEDV strain*	2010 Southern China	Unknown	Bat (*Scotophilus kuhlii*)	Swine farm	(132)
	Porcine deltacoronavirus (PDCoV)	2009 Hong Kong	Asian leopard cat (*Prionailurus bengalensis*)? Ferret badger (*Melogale moschata*)?	Avian, sparrow and bulbul	Illegal live-animal market?	(132, 138–140)
	Swine acute diarrhea syndrome coronavirus (SADS-CoV)	2016 Guangdong, China	Unknown	Bat (*Rhinolophus* spp.)	Swine farm	(141, 156)
Dog	Canine respiratory coronavirus (CRCoV)	2003 United Kingdom	Cattle (BCoV)	Rodents? Bats?	Kennel	(10, 17, 106, 115, 116, 118)
	Canine enteric coronavirus (CCoV)	1971 Germany	Unknown	Bat (*Rhinolophus* spp.?)	Military dog kennel	(10, 115, 126, 156, 158)
	*Pantropic CCoV-IIa*	2005 Italy	Unknown	Bat (*Rhinolophus* spp.?)	Pet shop	(10, 115, 120, 126)
Cat	Feline coronavirus (FCoV)	1963 United States	FCoV-I: Unknown FCoV-II: Cat and/or dog (FCoV-I × CCoV-II)	Bat (*Rhinolophus* spp.?)	Shelters and catteries	(10, 127, 149)
	*Horizontally-transmitted FIP FCoV-II*	2011 Taiwan	Cat and/or dog (FCoV-I × CCoV-II)	Bat (*Rhinolophus* spp.?)	Shelter	(10, 127, 144)

*The entry “Unknown” may either suggest that an intermediate spillover host exists but it has not been identified, or that it may not exist. Question marks represent uncertainty*.

*FCoV-I × CCoV-II denotes recombination between FCoV-I and CCoV-II*.

Additionally, animals kept under poor conditions or exposed to stress (e.g., during transport) suffer from poor health and suppressed immune systems, rendering them more susceptible to infections (64, 160). For example, mink fur farms, where animals are usually kept in small, unhygienic enclosures, generated new strains of SARS-CoV-2 causing secondary zoonoses (95, 101–103). The wildlife trade and wet markets are conducive to disease emergence as well, since animals are transported and kept in small, unhygienic cages next to many different animal species (160). Indeed, a study showed that civets in markets were disproportionately positive for SARS-CoV-1 compared to civets on the supplying farms (30). Further, SARS-CoV-1 isolates from a civet and a racoon dog at the same market, but from different regions of China, had an identical S-gene sequence, which differed from that of the other civet isolates, indicating the occurrence of cross-species transmission at the market (26). Accordingly, the concept of One Health is important for suppressing coronavirus emergence.

Little is still known about host ranges and cross-species transmissions of coronaviruses. Most studies on this topic have been motivated by finding appropriate animal models for vaccine development, or identifying potential host species enabling viral persistence. However, future studies should expand their surveys beyond domestic, captive, or common laboratory animals for a fuller comprehension of coronavirus emergence and the extent of its radiation (Figure 1A). Surveillance efforts of coronaviruses in the wild are underway (e.g., PREDICT, Global Virome Genome) (161, 162), which are important for identifying new coronaviruses with zoonotic potential [reviewed in (163)], tracking spillover pathways, and potentially filling in the host range gaps of known coronaviruses in humans and domestic mammals.

Concurrently with the global expansion of humans and domestic mammals, various coronaviruses have emerged as a result of cross-species transmission among humans, and domestic and wild animals. Conceivably, the human and domestic mammal population increase yielded a large enough susceptible population to maintain coronavirus circulation, provided more opportunities for novel coronavirus emergence via spillover among different species, and brought humans and domestic animals in closer contact with wild reservoirs (164–166). The mechanisms governing the surge and radiation of these recently emerged coronaviruses require further investigation. Actions reducing people's dependency on domestic animals and animal products, while improving the health of the animals remaining in captivity, may mitigate coronavirus emergence.

## Author Contributions

NN performed the literature review and wrote the manuscript.

## Funding

NN was supported by the Philanthropic Educational Organization (P.E.O.) Scholar Award from the International Chapter of the P.E.O. Sisterhood, the Environmental Venture Project Grant from the Stanford Woods Institute for the Environment, the Stanford Data Science Scholars program, and the Department of Biology at Stanford University.

## Conflict of Interest

The author declares that the research was conducted in the absence of any commercial or financial relationships that could be construed as a potential conflict of interest.

## Publisher's Note

All claims expressed in this article are solely those of the authors and do not necessarily represent those of their affiliated organizations, or those of the publisher, the editors and the reviewers. Any product that may be evaluated in this article, or claim that may be made by its manufacturer, is not guaranteed or endorsed by the publisher.
